# Prescriptive analytics applied to brace treatment for AIS: a pilot demonstration

**DOI:** 10.1186/1748-7161-10-S2-S13

**Published:** 2015-02-11

**Authors:** Eric Chalmers, Doug Hill, Vicky Zhao, Edmond Lou

**Affiliations:** 1 Electrical & Computer Engineering Department, University of Alberta, 9107 116 Street, Edmonton, Alberta, Canada, T6V 2V4; 2 Rehabilitation Research & Technology, Alberta Health Services, 10105 112 Ave, Edmonton, Alberta, Canada, T5G 0H1

## Abstract

**Background:**

Prescriptive analytics is a concept combining statistical and computer sciences to prescribe an optimal course of action, based on predictions of possible future events. In this simulation study we investigate using prescriptive analytics to recommend optimal in-brace corrections for braced Adolescent Idiopathic Scoliosis (AIS) patients. The objectives were to estimate the efficacy of these recommendations, ultimately working toward improved brace design protocols.

**Methods:**

Data was obtained for 90 AIS patients who had finished brace treatment at our center (60 full-time and 30 nighttime braces). Rates of ≥6 degree progression were 53% for daytime and 30% for nighttime braces. A modeling technique previously developed by our group was used to predict these patients’ likely treatment outcomes given a range of in-brace corrections – the model was blinded to the true outcomes during this process. Each patient’s ‘recommended’ correction was identified as the least aggressive correction resulting in a desirable predicted outcome.

The efficacy of these recommendations was estimated using a technique called “clinical trial simulation” (CTS). This technique used a statistical model to predict progression rate under the model-recommended treatment, and compared it to the true progression rate, observed retrospectively, under the actual treatment. Significance was calculated using a permutation test.

**Results:**

Model-recommended corrections ranged from 20%-58% for daytime and 65%-130% for nighttime braces, roughly corresponding with previous literature. Interestingly, in 37% of cases the recommended correction was less than that which had actually been applied, suggesting some opportunity for less aggressive (more comfortable) braces without compromising treatment outcome.

The CTS estimated 26% fewer progressive cases using the model-recommended in-brace correction, over the actual correction observed retrospectively in the charts (p=0.05). The patients whose correction decreased under the model’s recommendation did not show an increased progression rate.

**Conclusions:**

Optimal correction may be less than the maximum achievable correction. The preliminary results suggest that considering model-generated recommendations during brace fitting could improve outcomes. Future work will expand the system to recommend wear-times as well as corrections, improving its clinical relevance. We envision this pilot demonstration to promote development of model-based decision support in scoliosis treatment, and prompt discussion on its future role.

## Background

Brace treatment is the most common non-surgical treatment for Adolescent Idiopathic Scoliosis (AIS). A brace designer usually tries to achieve the maximum possible in-brace curve correction, since correction is associated with successful treatment outcome [1-3]. Often the target is 50% correction. However each patient has unique demographic and clinical characteristics; we hypothesize that customizing brace treatment protocols per-patient would improve overall treatment results.

This pilot simulation studied the effect of applying customized in-brace corrections to each patient. Prescriptive analytics – a paradigm which combines statistical and computer sciences to prescribe an optimal course of action, based on analysis of past data [4] – was used. Prescriptive analytics uses a computer model to predict the result of each possible action, and then recommends the action giving the best predicted result. Chi *et al*. applied this concept to hospital selection [5] and heart disease risk reduction [6]. Tan *et al*. proposed using it to optimize protocols for reducing obesity [7]. Here the goal is to identify patient-specific “optimal” in-brace corrections [8].

## Methods

A previously designed predictive modeling technique [9] was used in this study. The technique considers three treatment outcome categories: progression, neutral, and improvement. It uses fuzzy logic to predict a patient’s “membership” in each category based on start-of-treatment measurements. These memberships are conceptually analogous to probabilities that the patient’s major Cobb angle will progress, improve, or remain neutral during treatment. The start-of-treatment measurements used by the technique are patient age, Cobb angle, scoliometer measurement, and in-brace correction. See [9] for details.

**Figure 1:**
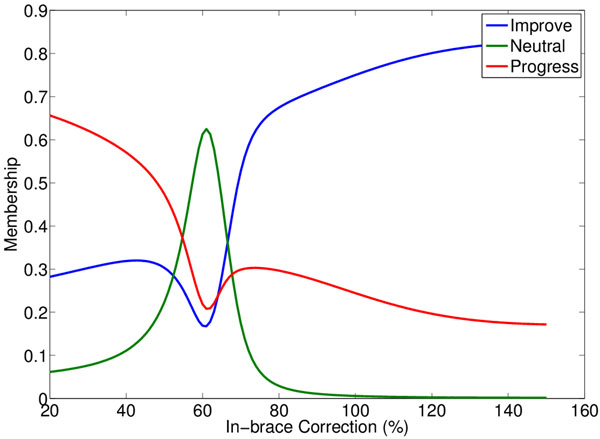
**Sample treatment outcome predictions.** Sample progress, neutral, and improve membership predictions for a particular patient, over a range of in-brace corrections. “Membership” is conceptually similar to probability; a patient’s “progression membership” is analogous to their probability of progression.

Records were obtained for 90 AIS patients who had finished brace treatment at our center from 2006-2013. The health research ethics board (Health Panel, University of Alberta) approved the study. All complete records from patients meeting the SRS criteria for bracing were used. Table 1 describes the patient sample. The aforementioned modeling technique was used to predict treatment outcome for each patient (this process was blinded to the true outcomes). In-brace corrections from 20% to 160% were considered, and for each patient the model predicted outcome for each correction in this range. A sample result is shown in figure 1: the three curves show how predicted progression, neutral, and improvement memberships change with varying in-brace correction.

**Table 1:** Patient Sample

Patient Measurement	Distribution
Age	13.4 ± 1.7 yrs

Sex	75 F, 15 M

Brace type	60 full-time TLSO 30 nighttime Providence

Major Cobb angle	30 ± 7°

Major curve apex	T11 ± 3 vertebrae

Curve type	63 single, 27 double

In-brace correction	TLSO: 39 ± 25% Providence: 113 ± 38%

Cobb progression at treatment finish	5 ± 10° 41 cases >5°

Characteristics of patient sample used in this study. Measurements were taken at the start of brace treatment.

These progress, neutral, and improvement predictions were used to identify suitable in-brace correction targets for each patient. Consider figure 1: at 60% correction the predicted neutral membership is at its peak, and the progress membership is relatively low. Thus 60% correction may be a good target correction for this patient as predictions indicate their curve would likely remain neutral. A night brace might attempt more correction, but figure 1 indicates a point of diminishing returns in the improve membership at about 80% correction; thus correction above 80% may be unnecessary for this patient.

**Figure 2:**
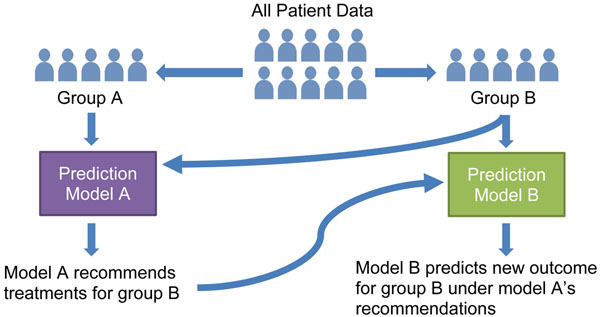
**Clinical trial simulation.** Clinical trial simulation procedure. Model A recommends in-brace corrections for patients in group B, with Model B predicting the recommendations’ effect. Predicted outcomes given the recommendations were compared to outcomes in the group B patients’ charts.

A clinical trial simulation (CTS) technique proposed by Chi *et al*. [6,10] and illustrated in figure 2 was used to test the efficacy of these in-brace correction recommendations. The 90 patient records were randomly divided into 2 equally-sized groups: A and B. Separate predictive models were developed using the data from each group. Model A produced in-brace correction recommendations for the patients in group B using the procedure described above. Model B then predicted the new treatment outcomes for these patients given model A’s recommendations. Thus, the CTS simulated a study in which recommended corrections were applied to group B, and the original group B patients served as matched controls. The predicted outcomes for group B under recommended in-brace correction were compared to the actual outcomes recorded in the patients’ charts. A CTS provides an unbiased estimation of the recommendations’ effect by training and using the two prediction models independently.

Overall progression rates from the patient charts were compared to (predicted) progression rates under the recommended in-brace corrections; the difference in progression rate was measured. Progression was defined as a >5° increase in Cobb angle by the end of treatment [11]. A permutation test calculated the confidence interval of the change in progression rate, based on the prediction model’s negative/positive predictive values.

## Results

Computer-generated in-brace correction recommendations ranged from 20%-58% for full-time braces, and 65%-130% for nighttime. In 37% of cases the recommended in-brace correction was lower than the actual correction which had been applied clinically, as recorded in the patient charts.

The CTS estimated 26% fewer progressions under the recommended in-brace correction, compared to the actual correction in patients’ charts. The 95% confidence interval ranged from 48% fewer to 4% more progressions. The estimated decrease in progression rate was statistically significant at p=0.05.

## Discussion

The CTS estimated that the computer-recommended in-brace corrections could reduce progressive cases by 26%. However it is unclear whether the recommended corrections would actually be achievable in practice: overall the recommendations agree with literature and corrections observed at our clinic, but some individual patients with stiff curves may not be capable of large corrections. Thus, what is perhaps most interesting is that many recommended corrections were *lower* than that actually applied. This may suggest some potential to build less aggressive (more comfortable) braces without compromising treatment outcome.

This study was performed using data from one center, and its limitations may affect the results’ generalization to other centers. Our patient sample was somewhat small, and involves two different brace types built by two orthotists. The patients’ compliance with brace-wear was unknown. Our approach may have unpredictable results at other centers, or on different patient groups.

The results of this pilot demonstration are promising, but the work is in its early stages with a significant amount of work to be done. The system will be expanded to recommend wear-times as well as corrections, producing a range of correction/wear-time combinations likely to result in success. This will be useful in cases where the “optimal” correction is not actually achievable. Also, this preliminary work used a clinical trial simulation; ultimately a prospective clinical trial will be necessary to prove clinical value.

## Conclusions

This preliminary study suggests that computer-generated recommendations may improve treatment outcomes, and may safely reduce aggressiveness of treatment in some cases.

## Competing interests

The authors declare that they have no competing interests.

## Authors' contributions

EC conceived of and carried out the study. EC, DH, VZ and EL assisted with manuscript preparation. All authors have approved the final manuscript.

## Declarations

Publication charges for this collection were funded by IRSSD 2014 Sapporo.

This article has been published as part of *Scoliosis* Volume 10 Supplement 2, 2015: Research into Spinal Deformities 9: Short Papers from the IRSSD 2014 Meeting. The full contents of the supplement are available online at http://www.scoliosisjournal.com/supplements/10/S2.
